# A descriptive, cross-sectional study of medical student preferences for vodcast design, format and pedagogical approach

**DOI:** 10.1186/s12909-017-0926-z

**Published:** 2017-05-19

**Authors:** Robin K. Pettit, Marjorie Kinney, Lise McCoy

**Affiliations:** 0000 0004 0383 094X grid.251612.3 A. T. Still University, School of Osteopathic Medicine in Arizona, 5850 E. Still Circle, Mesa, AZ 85206 USA

**Keywords:** Vodcast, Video podcast, Distance education, Vodcast multiplication factor, Vodcast attributes, Vodcast length, Transcript

## Abstract

**Background:**

Vodcasts (video podcasts) are becoming increasingly popular in medical education. At A.T. Still University School of Osteopathic Medicine in Arizona (ATSU SOMA), vodcasts are an essential component of our blended learning environment, where year 2–4 students train in a contextual setting at community health centers across the U.S. Vodcasts are used far less frequently in our year 1 residential learning environment at the main campus in Arizona, but we are considering moving to significantly more interactive educational experiences with on-demand videos followed by in-class activities. The aim of this study was to determine stakeholder (i.e. medical student) preferences for vodcast design, format, and pedagogical strategies. The overall goal was to increase opportunities for students to learn with this modality.

**Methods:**

An interactive Qualtrics™ survey was administered to three cohorts of medical students. The survey generated quantitative and open-ended response data that addressed principles of vodcast instructional design and learning. Responses to survey items were analyzed for statistical significance using the independent samples t-test for interval data, the chi-square test for categorical data, and the Kruskal-Wallis test for ordinal data, using the post-hoc Bonferroni procedure to determine the appropriate α level. Responses to open-ended prompts were categorized using open- and axial-coding.

**Results:**

The most highly valued vodcast attributes, considered essential by all three cohorts, were clear explanations, organization, conciseness, high-yield for medical board exams, and the ability to speed vodcasts up. The least helpful vodcast attributes for all three cohorts were music and objects moving on screen. The average preferred vodcast length for each cohort was 27–28 min. There were significant differences between the less experienced learners in the residential setting and the more mature learners in the blended learning environment regarding certain vodcast attribute preferences, format of included practice questions, explanations for preferred vodcast lengths, and reasons for not viewing vodcasts.

**Conclusions:**

Overall, learner preferences were in line with non-interactive, screen-capture type vodcasts, which have lower demands on institutional cost and faculty production time than Flash™-type interactive vodcasts. Students in the blended learning environment were much more focused on vodcast features that decreased their time commitment, including a preference for noninteractive vodcasts. Given the increase in distance learning in medical education, our results should be of value to other medical programs.

**Electronic supplementary material:**

The online version of this article (doi:10.1186/s12909-017-0926-z) contains supplementary material, which is available to authorized users.

## Background

Vodcasts (video podcasts) are gaining in popularity in medical education [1–9]. Videos meet the needs of the current digital generation of students [10, 11], offering advantages such as convenience, ubiquity of access, ability to self-pace, and ability to repeat content [3, 12]. For instructors, vodcasts allow standardization and potential modularization of teaching materials, and dissemination to learners at different locations. Randomized controlled trials with undergraduate medical students have demonstrated that learning gains are similar when vodcasts and typical lecture sessions are compared [7, 13].

At A.T. Still University School of Osteopathic Medicine in Arizona (ATSU SOMA), year 1 osteopathic medical students (OMS1s) receive instruction at the Mesa, AZ campus (residential learning environment), while year 2–4 students train in a contextual setting at and around one of twelve community campuses nationwide (blended, service learning environment) [14, 15]. At these community health centers (CHCs), year 2 osteopathic medical students (OMS2s) receive most of their didactic content from Mesa-based basic science and clinical faculty asynchronously, via vodcasts, with in-person instruction occurring weekly from Regional Directors of Medical Education at each site. As such, vodcasts are a critical instructional component of the distant, blended learning environment.

In contrast, vodcasts are used relatively infrequently in the residential learning environment at the Mesa campus. However, we are considering the possibility of moving to significantly more flipped content. In a flipped classroom, typical lecture and homework elements are reversed; video lectures are viewed by students prior to class, while in-class time is devoted to more student-centered activities [16].

SOMA faculty currently have autonomy in selecting vodcast software that best fits their technical background and teaching goals. As such, students receive a variety of formats and presentation styles. Table 1 summarizes the attributes of vodcast software used by SOMA faculty. Vodcast software is either non-physically interactive screen-capture (mp4 output) or Flash™ (HTML5/Flash output), and the options vary in cost, ease of use, and production features. For example, with Flash™-type vodcast software, the user can physically interact with the content, often an included practice quiz. However, with this software, the user cannot control the speed of the vodcast, and the mouse/cursor is not visible to the user. As shown in Table 1, the majority of SOMA vodcasts are recorded and produced with the screen-capture software Camtasia™. With screen-capture software, the user cannot physically interact with the content, but they can control the speed of the vodcast, and the instructor’s mouse/cursor is visible.

**Table 1:** Vodcast recording and production software used by SOMA faculty and their attributes

Recording software	Production software^a^	Number of faculty using^b^	Approximate cost of software/user^c^	Output format faculty provides to student^d^	Screen-capture (sc) or flash	Ability of student to control speed	Ability of student to interact with content via mouse	Potential for table of contents	Presenter’s mouse/cursor appears on screen
TechSmith Camtasia	TechSmith Camtasia	29	$ (for Mac); $$ (for PC)	mp4	sc	yes	no	no; yes for Camtasia Studio (PC) if published as flash (.swf or .flv)	yes
Adobe Presenter	TechSmith Camtasia	1	$$ + $	mp4		yes	no	yes but won’t be interactive	no
Articulate Presenter	TechSmith Camtasia	2	$$$ + $	mp4		yes	no	yes but won’t be interactive	no
PowerPoint (for PC)	TechSmith Camtasia	1	$ + $	mp4	sc	yes	no	no	yes
PowerPoint (for Mac)	PowerPoint (for Mac)	2	$	mp4	sc	yes	no	no	yes
Shineywhitebox iShowU	Apple GarageBand	1	$	mp4	sc	yes	no	no	yes
Ambrosia Snapz Pro	Apple Garage Band (plus iMovie for editing)	1	$	mp4	sc	yes	no	no	yes
Telestream Screenflow	Telestream Screenflow	1	$	mp4	sc	yes	no	no	yes
Araleium Screenflick	Araleium Screenflick	1	$	mp4	sc	yes	no	no	yes
Adobe Presenter	Adobe Presenter	4	$$	HTML5/flash	flash	no	yes	yes	no
Adobe Captivate	Adobe Captivate	3	$$	HTML5/flash	flash	no	yes	yes	no
Articulate Storyline	Articulate Storyline	4	$$$	HTML5/flash	flash	no	yes	yes	no
Articulate Engage	Articulate Engage	1	$$$	HTML5/flash	flash	no	yes	yes	no

^a^The same 3–4 faculty use flash Adobe Presenter, Adobe Captivate, Articulate Storyline

^b^A few faculty use more than one

^c^$ = <100; $$= > 100; $$$= > 1000

^d^Some of these have optional outputs

Our reliance on vodcasts for the majority of the blended learning environment instruction, and a potentially increased proportion of residential learning environment instruction, indicated a need to determine stakeholder (i.e. medical student) preferences for vodcast design, format, and pedagogical strategies. The stakeholders differed in both time in medical school and current learning environment. The goal was to use this information to guide best practices in vodcast creation and production in order to facilitate student learning. The study comprises part of an ATSU SOMA goal to improve technology-enhanced learning in our curriculum. The following two research questions were addressed: Are vodcast preferences for students in residential and distant, blended learning environments different? Can analysis of preferences guide improvements in vodcast creation and production?

## Methods

### Participants and setting

This descriptive, cross-sectional study was conducted in January, 2016 at ATSU SOMA during the 2015–2016 academic year, with three cohorts of medical students: 105 first-year medical students (Class of 2019), 109 second-year medical students (Class of 2018), and 100 third-year medical students (Class of 2017). Year 1 students were at the Mesa campus; year 2 and year 3 students were at their respective CHCs.

The curriculum for the three student cohorts remained consistent, except that the first course in the academic year for the Class of 2019 (OMS1s) was a pilot 3-week anatomy course, Basic Structural Foundations, where 75% of the content was flipped. The other 25% was lecture-based with activities. For flipped sessions, students viewed vodcasts containing interactive quizzes prior to coming to class for practice activities. All vodcasts for Basic Structural Foundations were recorded and produced with Adobe Presenter™ or Adobe Captivate™ (Table 1), and transcripts were provided with all vodcasts. The remainder of the year 1 curriculum for the Class of 2019 was primarily lecture based, approximately 70% of these with built-in activities, and a small percent (5%) flipped with vodcasts. The Class of 2018 (OMS2s) and the Class of 2017 (OMS3s) had previously experienced the same primarily lecture-based curriculum, and did not experience the pilot course.

The ATSU Institutional Review Board deemed the study exempt from Institutional Review Board reporting requirements for human subjects research.

### Development of the survey instrument

An original, eight-item, interactive, electronic questionnaire was used to gather student feedback on vodcasts (Additional file 1). To develop the Qualtrics™ survey, authors reviewed the multimedia learning literature, and developed items to investigate domains related to learning, motivation and instructional design [17–23]. In addition, survey items and language were built on 4 years of internal exploratory studies and needs assessments conducted by the SOMA Technology-Enhanced Active Learning for Medical Education committee. These studies included an informal, retrospective review of student course feedback related to vodcasts, faculty input regarding their vodcast training needs, and the development of basic vodcast guidelines for faculty in 2014. The entire research team tested the survey several times before use to fine-tune the associated language.

Three questions at the beginning of the survey addressed age range, gender and level in the program. One of the interactive items was a drag-and-drop question (*Drag and drop each of the following vodcast attributes into the box that best describes their value to your learning: Essential, Nice to have, or Not helpful*), and the other a sliding scale question (*Slide the scale to indicate your ideal vodcast length. Slide the dial to any number of minutes between 1 and 60*). One of the open-ended questions solicited information on students’ pet peeves regarding vodcasts; another probed students’ favored external (outside of SOMA) resource for vodcasts. Non-open-ended questions investigated explanations for ideal vodcast lengths, preferred format for incorporation of practice questions, reasons for not viewing vodcasts, and students’ preferred devices for playing vodcasts.

### Data collection

Survey data collection involved an email solicitation containing a clickable link to an online survey. All students were surveyed at the beginning of January, 2016, the week after returning from winter break. As detailed below, at the time of the survey, the three cohorts had experienced medical school for one semester (OMS1), three semesters (OMS2) or five semesters (OMS3). Thus, both learning environment and maturity as learners may have influenced survey responses.

Year 1 students (Class of 2019) received the email survey during an unrelated large group session given by a faculty member who was not involved in the research. The students were given approximately 10 min to complete the survey on their personal devices during this class. The year 1 survey closed 4 days later.

Year 2 students received the email survey at their respective CHCs. They were sent three email reminders and the survey closed early February, 2016. This was the beginning of the second semester at their CHCs; as such, these students had experienced one semester of didactic instruction via vodcasts, in addition to their first year of instruction at the Mesa campus.

Year 3 students received the email survey at their respective CHCs. They were sent three email reminders and the survey closed early February, 2016. In addition to their first year of instruction at the Mesa campus, these students had experienced an entire year of didactic instruction via vodcasts (their 2nd year).

Survey participation was voluntary and anonymous. Students were not asked to provide evidence of completion, and there were no rewards offered for completing the survey.

### Data analysis

Statistical analyses were completed using the statistical analysis software IBM SPSS Statistics 23™. Independent samples t tests were conducted to determine statistical significance between respondents in various participant groups for interval data (vodcast length). Chi-Square tests were conducted to determine statistical significance between respondents in the three participant groups for categorical data (preferred practice quiz scenarios, scenarios for not viewing vodcasts, reasons for preferred vodcast length). Kruskal-Wallis tests were conducted to determine statistical significance between respondents in the three participant groups for ordinal data (value of vodcast attributes). When differences existed using these methods, pair-wise post-hoc comparisons using the Bonferroni procedure were completed to reduce the chance for Type I errors.

We used open- and axial-coding [24] to analyze student responses to open-ended survey prompts. The process of coding the narrative data began with the first researcher categorizing student comments into codes. Each student comment was kept intact, as opposed to breaking it down further into sub-points. The first researcher annotated the margins of the table with memos and questions to the second researcher. After considering the themes and questions that emerged from the data, the second researcher edited some of the codes, and suggested re-coding a few of the comments. When disagreements arose, we reviewed learning theory and delineated distinctive features of each code. We debated disagreements freely until reaching consensus. In a few instances, we sought inter-coder confirmation from the third researcher. Finally, the entire research team reviewed all of the completed tables and their text summaries.

## Results

A total of 221 respondents completed the vodcast survey. The demographics and response rates of each cohort are summarized in Table 2.

**Table 2:** Response rates and respondent demographics for the Class of 2017, Class of 2018 and Class of 2019

Class	Number of respondents	Response rate by class	Gender^a^		Age^a^			
(year of program)			M	F	20–25	26–30	31–35	36–40
2019 (OMS1s)	104	99.00%	54	50	65	31	7	1
2018 (OMS2s)	70	64.20%	35	34	24	34	11	0
2017 (OMS3s)	47	47.00%	24	23	10	31	4	2

^a^One student in the Class of 2018 did not specify gender or age.

### Prioritizing preferred vodcast attributes

**Fig. 1:**
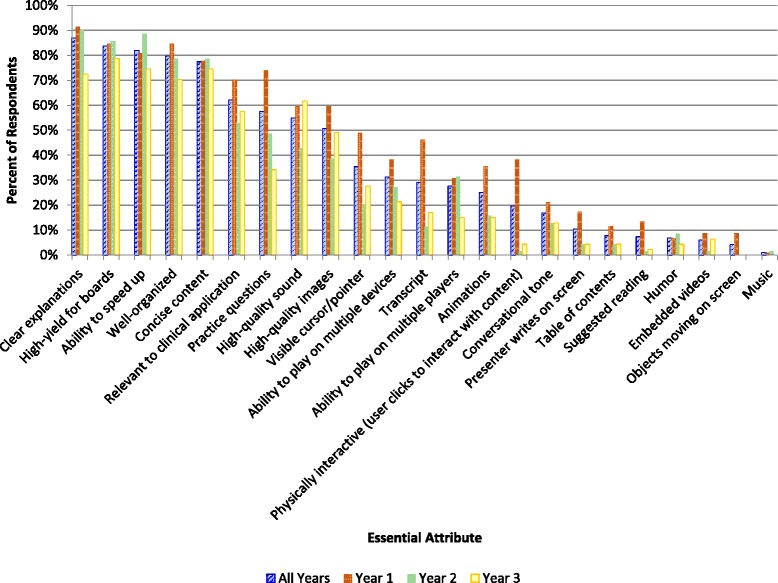
Summary of student responses to the prompt *Drag and drop each of the following vodcast attributes into the box that best describes their value to your learning: Essential, Nice to have, or Not helpful.* This figure shows the relative ranking of attributes that students deemed *Essential*. Graphs summarizing the relative student ranking of *Not helpful* and *Nice to have* attributes are in Additional files 2 and 3. On the far right side of the x axis, an interactive *Table of contents* is generated automatically with the Flash™ vodcast software listed in Table 1. *Suggested reading* refers to slides with assigned pages/chapters in required/recommended textbooks. *Objects moving on screen* includes animations, PowerPoint™ slide transitions and the mouse/cursor

The first survey item was a drag-and-drop task that queried the learning/instructional design value of 23 vodcast attributes. The attributes were simply alphabetized, and not in any other order or categories so as not to bias responders. Students were asked to distribute the attributes into one of three boxes labeled *Essential, Nice to Have* and *Not Helpful*. The most highly valued vodcast attributes, rated *Essential* by nearly all responders in all three cohorts, were *Clear explanations, High-yield for boards, Ability to speed up, Well-organized,* and *Concise content* (Fig. 1). *Relevant to clinical applications* and *Practice questions* were two other highly valued vodcast attributes (Fig. 1). *High quality sound* and *High quality images* were the next most highly valued attributes for all three cohorts, with only a handful of students rating them as *Not Helpful* (Fig. 1, Additional file 2). For the three cohorts combined, *Music*, *Objects moving on screen, Table of contents* and *Suggested reading* were the four vodcast attributes most frequently rated as *Not Helpful* (Additional file 2)*.* Several attributes clearly stood out as *Not Helpful* for OMS2,3s (Additional file 2). In particular, the attribute *Physically interactive* (*user clicks to interact with content*) was much more highly valued by OMS1s than OMS2,3s (Fig. 1, Additional file 3) [Chi-Square Test, *p* < 0.001, post-hoc Bonferroni-adjusted required α <0.0056].

### Practice questions

**Fig. 2:**
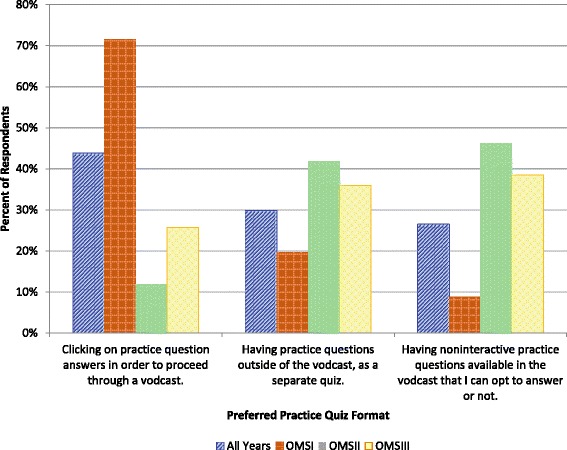
Summary of student responses to the prompt *Which of the following scenarios regarding practice quiz questions within vodcasts do you prefer?*

Overall, practice quiz questions were highly valued, but they were significantly more likely to be rated *Essential* by OMS1s than by OMS2s (*p* < 0.005 - post-hoc Bonferroni-adjusted required α <0.017), and by OMS2s than by OMS3s (*p* < 0.005 - post-hoc Bonferroni-adjusted required α <0.017) (Fig. 1). Practice quiz questions can be provided within vodcasts, and depending on the software, can be physically interactive or non-physically interactive. With physically interactive questions, the user must stop and click a button with their mouse or enter a response in order to proceed through a vodcast. For non-physically interactive questions, the user can pause and work through a question, or continue, and perhaps work on the question later. Another option is to provide practice questions as a separate file. At SOMA, there are faculty that use each of these methods. Responses to the prompt *Which of the following scenarios regarding practice quiz questions within vodcasts do you prefer?* are summarized in Fig. 2. There was a statistically significant difference between the responses of OMS1s and OMS2,3s, with OMS1s overwhelmingly preferring physically interactive questions (*Clicking in order to proceed* rated more highly by OMS1s *p* < 0.001, *Having practice questions separate from vodcast* and *Noninteractive questions within vodcast* rated more highly by OMS2s and OMS3s *p* < 0.001 and *p* < 0.005, respectively - post-hoc Bonferroni-adjusted required α <0.017).

### Vodcast pet peeves

We intentionally solicited negative feedback with the open-ended prompt *What is your biggest pet peeve regarding vodcasts?,* because of its value to improving vodcast creation and production. Responses were categorized by theme and sub-theme using open- and axial-coding [24]. Eight key themes were identified: Audio, Video, Can’t control speed, Other technical issues, Pace/Length, Content quality, No transcript, and Scheduling (Table 3). The most common student comment under the theme Audio, subtheme Poor sound quality, was “too quiet”. Other responses associated with Audio were informative about noises that can be distracting for students, including “cell phones ringing” (subtheme Ambient noise). The most frequent comment under Video was “poor image quality” (subtheme Images). Another important theme was Can’t control speed, and almost all of the comments were from OMS1s, including “I don’t like [it] when presenters use the Adobe program for their vodcasts because then you don’t have the ability to speed up or slow down the lectures.” Most of the comments associated with Pace/Length were about long vodcasts (subtheme Length), and some provided insight; for example, “when they are too long lose interest”. Professor-specific concerns were categorized under the theme Content quality. Comments associated with Content quality, subtheme Distractions, were helpful, including “writing all over the pictures”; “overly cluttered slides”; and “too many pointless flashy visual transitions in a Powerpoint”. Another subtheme for Content quality, Lack of conciseness, was clearly more important to students in the blended learning environment, with nearly 90% of the comments from OMS2,3s. No transcript was a pet peeve for nine OMS1s, but no OMS2,3s. Similarly, Scheduling (e.g. “the time allotted is sometimes inadequate for the actual time it requires to thoroughly learn from the vodcast and not simply just watch it*”*) was a pet peeve for three OMS1s, but no OMS2,3s. In the blended learning environment at the CHCs, there is no set schedule for viewing the vodcasts assigned each week.

**Table 3:** Summary of student responses to the open-ended prompt *What is your biggest pet peeve regarding vodcasts?*

Theme	Subtheme (where applicable)	Number of student comments	Percent of student comments
Audio	Poor sound quality Ambient noise Fluency	24 6 4	11% 3% 2%
Video	Poor video quality Images	8 7	4% 3%
Can’t control speed		20	9%
Other technical issues	Not playing File size Platform	11 3 2	5% 1% 1%
Pace/Length	Pace Segmentation Length	2 2 16	1% 1% 7%
Content quality	Organization Explanations Mismatch Lack of conciseness Distractions Outdated Reading the slides Lack of interaction	21 9 6 19 8 10 9 8	10% 4% 3% 9% 4% 5% 4% 4%
No transcript		9	4%
Scheduling		3	1%
No pet peeve		7	3%

### Ideal vodcast length

**Fig. 3:**
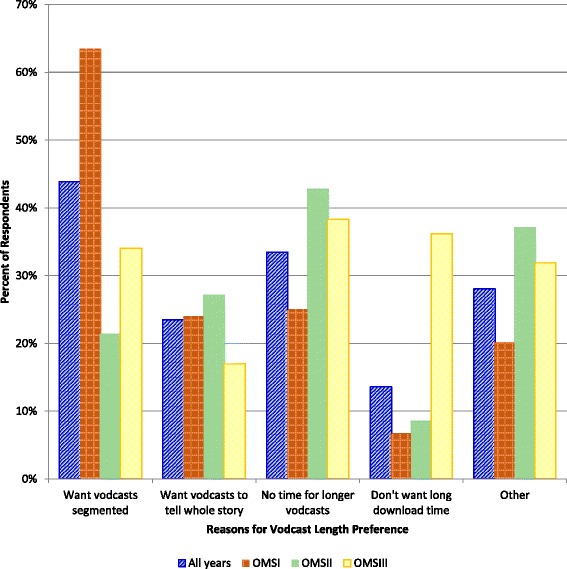
Summary of student responses to the ideal vodcast length follow-up prompt *I prefer this length because*…

While length preferences ranged from 10 to 60 min, most students preferred lengths between 15 and 30 min (mean = 27.6, standard deviation = 10.1). The average ideal vodcast length for all three cohorts was 27–28 min. There were no statistically significant differences between the distribution of the preferred lengths between the cohorts (Kruskal-Wallis, *p* = 0.751). Responses to the follow-up question *I prefer this length because (Check all that apply)*…are summarized in Fig. 3. Significantly more OMS1s than OMS2s and OMS3s said their preferred length was because they *want vodcasts segmented* (*p* < 0.001 for OMS1 vs. OMS2 and *p* < 0.005 for OMS1 vs. OMS3 - post-hoc Bonferroni-adjusted required α <0.017*)*. Significantly more OMS2s and OMS3s than OMS1s said their preferred length was because they have *no time for longer vodcasts* (*p* < 0.010 for OMS1 vs. OMS2 and *p* < 0.001 for OMS1 vs. OMS3 - post-hoc Bonferroni-adjusted required α <0.017*)*. OMS3s were much more likely than OMS1s and OMS2s to choose *don’t want a long download time* (*p* < 0.001 for OMS1 vs. OMS3 and *p* < 0.001 for OMS2 vs. OMS3 - post-hoc Bonferroni-adjusted required α <0.017*)*. The 5th choice for this prompt was *Other. Please explain*, and these open-ended comments (62 respondents) provided additional insight into individual selections (Additional file 4).

### Reasons for not viewing vodcasts

**Fig. 4:**
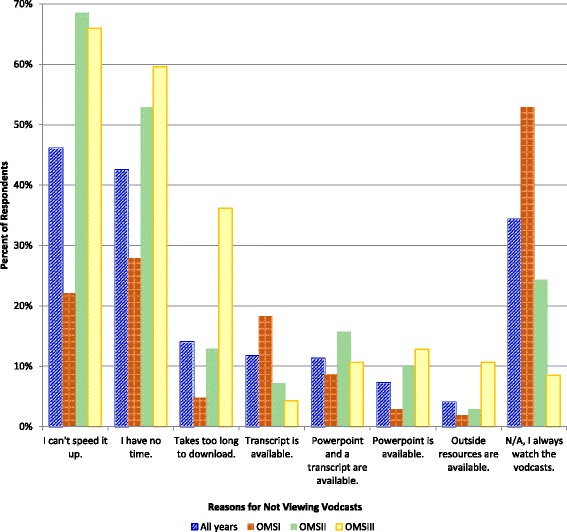
Summary of student responses to the prompt *I am less likely to view a vodcast when*…

Does the lack of a particular vodcast attribute or a vodcast pet peeve potentially result in students not viewing required vodcasts? When prompted with *I am less likely to view a vodcast when (Check all that apply)*…the most common reasons for all three cohorts were *I can’t speed it up*, *I have no time,* and *Takes too long to download* (Fig. 4). The OMS2s and OMS3s were statistically significantly more likely to rate the attributes *I can’t speed it up*, *I have no time* and *Takes too long to download* as reasons for not viewing vodcasts (*p* < 0.001 for *I can’t speed it up* and *I have no time,* for OMS1s vs. OMS2s and OMS1s vs. OMS 3s and *p* < 0.001 for *Takes too long to download* for OMS1s vs OMS3s - post-hoc Bonferroni-adjusted required α <0.017 for all). OMS1s were much more likely than OMS3s to select when a *transcript is available* as a reason for not viewing required vodcasts (*p* < 0.05 for OMS1s vs. OMS2s and *p* < 0.010 for OMS1s vs. OMS3s - post-hoc Bonferroni-adjusted required α <0.017).

### Favored external resources

A few students indicated that they were less likely to view a vodcast when outside resources were available (Fig. 4), and those resources could include vodcasts. Responses to the open-ended prompt *What is your favored external (outside of SOMA) resource for vodcasts?* are summarized in Additional file 5. YouTube and Khan Academy were popular for all three cohorts. For OMS2s and OMS3s, Pathoma and Doctors in Training vodcasts were popular as well.

### Preferred device

By far, the preferred device for playing vodcasts was laptop computers, particularly Macintosh laptops (Table 4). A few students selected desktops or iPads, and even fewer their iPhone or a tablet. There were three responses to the prompt *Other, Please explain* in this section of the survey: *Surface Pro; PC and MAC*; and *then I write my notes on my iPad as I watch.*

**Table 4:** Summary of student responses to the prompt *Which device do you prefer to play vodcasts on?*

Preferred device for playing vodcasts	Number of respondents		
	OMS1	OMS2	OMS3
Macintosh laptop	55	47	18
PC laptop	32	12	14
PC desktop	6	6	1
iPad	4	1	4
Macintosh desktop	2	0	1
iPhone	0	1	1
Android tablet	0	0	1
Android phone	0	0	0
iPod	0	0	0

## Discussion

### Are vodcast preferences for students in residential and distant, blended learning environments different?

Key vodcast themes that emerged for both the less experienced learners in the residential environment and the more mature learners in the blended learning environment were a desire for organization, clear explanations, conciseness, high-yield board content, user-controlled speed, and a length between 15 and 30 min. The vodcast attributes *Well-organized* and *Clear explanations* may help students mentally organize the presented material into a coherent structure and integrate the presented material with existing knowledge, reflecting two of the three learning principles of multimedia learning [22]. The attribute *Concise content* likely facilitates selecting, or attending to the relevant incoming material, the third learning principle of multimedia learning [22]. The high ranking of *High-yield for boards* (i.e. United States Medical Licensing Examination and the Comprehensive Osteopathic Medical Licensing Examination of the United States) was not surprising since adults are more intrinsically motivated to complete learning tasks when they understand their full value and relevance to academic, workplace, or personal goals [25–27]. This also explains the high ranking of the attribute *Relevant to clinical application*.

Student preference for user-controlled speed was apparent with the high ranking of the attribute *Ability to speed up,* and the number of pet peeves associated with the theme Can’t speed up. With screen-capture, but not Flash™ vodcast software, the user can control the speed of the vodcast. A benefit of controlling the speed is that students can self-pace, for example fast-forwarding or replaying video sections as needed [10]. In addition, student engagement is known to increase as vodcast speaking rates increase [17, 19]. The speed at which a vodcast is played may also relate to level of difficulty and the number of times the student has viewed it.

The next most highly valued vodcast attribute overall was *Practice questions*. We have demonstrated in previous studies [28, 29] how much value our students place in practice questions as a formative method to prepare for summative exams. Indeed, there is evidence that self-testing while studying is an effective learning method [30]. In the current study, OMS1s placed more value in *Practice questions* than OMS2s and OMS3s, perhaps because OMS2s and OMS3s access more external resources for questions while they are studying for board exams, and they may become more adept at independently locating practice questions as they advance through the program.

Another difference regarding practice questions was how they were incorporated in vodcasts. Students in the residential learning environment (OMS1s) preferred physically interactive questions, and students in the blended learning environment (OMS2,3s) preferred non-physically interactive questions. Similarly, the attribute *Physically interactive (user clicks to interact with content)* was much more likely to be rated *Not helpful* than *Essential* by students in the blended learning environment. There are two possible explanations for these findings. There may be a novelty effect with the less experienced learners in the residential environment. A second possible explanation is that because OMS2s receive much of their instruction via vodcasts (average of 8.1 h of vodcasts/week; course averages range from 6.7 to 9.4 h/week), they may prioritize the ability to move through the material as rapidly as possible, and clicking through content may slow them down.

While the average preferred vodcast length was identical for students in both learning environments, explanations for preferences revealed differences. Students in the residential learning environment had a strong preference for segmented vodcasts, while time was a more critical factor for students in the blended learning environment. A common theme in open-ended responses to explain length preference was attention span, with some students reporting a 15-min attention span, and others up to 40 min. Similarly, a ‘brief’ vodcast for one student was an overly long vodcast for another. Other open-ended comments that provided insight into preferred vodcast length described longer vodcasts freezing up and longer vodcasts being more disorganized and less concise. Related to these perceptions, Guo et al. [19] proposed that shorter videos are engaging not only due to length, but also because they are better planned.

Length is arguably a leading reason that students might choose to not view a vodcast. The time required to view, pause, look things up, and take notes from a vodcast typically amounts to twice the length of the vodcast; in other words, a 20 min vodcast is roughly equivalent to a 40 min lecture (there were several student comments that referred to this ‘vodcast multiplication factor’). The most common options selected in response to *I am less likely to view a vodcast when*…were: *I can’t speed it up*, *I have no time,* and *takes too long to download*. These three response options were particularly evident for students in the blended learning environment. Embedded videos, which can increase vodcast length considerably, were much more frequently rated *Not Helpful* than *Essential,* especially by students in the blended learning environment. The relatively large number of vodcasts received by year 2 students may also help explain why OMS1s were much more likely to *always watch the vodcasts* than OMS2s and OMS3s. These more mature learners may have developed alternate methods of assimilating information in vodcasts, for example, by reading textbooks.

Provision of transcripts can result in students not viewing vodcasts, and this was more apparent for OMS1s than OMS2s and OMS3s. The explanation for this is likely that OMS2s and OMS3s did not have the pilot course Basic Structural Foundations, where transcripts were provided with all vodcasts. Very few faculty provide transcripts elsewhere in the curriculum. Students opting out of viewing vodcasts is potentially concerning because long-term memory is enhanced when students connect with information using multiple senses [31]. For multimedia learning specifically, learning is enhanced when both visual and auditory channels are stimulated [20, 21]. Back et al. [32] found that medical students had higher knowledge gains with vodcasts compared to textbooks, while Edmond et al. [33] showed that medical students had equivalent learning gains with vodcasts and written handouts. Many variables could contribute to these conflicting results, including the skill of the teacher and the difficulty of the topic. Ideally, students would use three learning modes if provided a transcript with each vodcast- listening, watching and reading. The transcript results have stimulated much discussion amongst our faculty, and we hope that with the increasing popularity of vodcasts in medical education, faculty at other institutions will soon join the conversation. Questions we are discussing include: Are students choosing to not view vodcasts to save time and/or because they perceive that they learn equally well by reading alone? Should transcripts be provided for learners who prefer reading, even though this could result in some students not viewing vodcasts? Are students who choose to only read transcripts at a disadvantage without the visual and verbal signaling cues in multimedia vodcasts? Should transcripts be withheld when they have the advantage of being readily transferrable into Microsoft OneNote™? Providing transcripts to students with relevant learning accommodations is, of course, justified.

### Some recommendations for improving vodcast creation and production

When developing vodcasts, it is worthwhile to consider those attributes students were more likely to rate *Essential* or *Not helpful,* no matter what level of medical student and what volume of vodcasts received. In addition, to facilitate learning and encourage viewing, efforts should be made to reduce cognitive load and increase engagement.

#### Reducing cognitive load

Weeding (removal of non-essential content), inclusion of signaling features, and segmentation are potential solutions to help learners select, organize and integrate content in vodcasts. Ibrahim et al. [34] showed that students who received videos that had been weeded, included signaling to direct students’ attention to relevant information, and incorporated breaks between segments, had better knowledge transfer, structural knowledge acquisition, and lower perceived levels of learning difficulty compared to students receiving videos without weeding, signaling and segmenting.

Weeding involves creating presentations that are as concise and coherent as possible because extraneous information, for example music or flashy animations, can cause students to engage in incidental processing [21]. Signaling can include stressing key words in speech, underlining, arrows, organizing text by adding outlines and headings, inclusion of learning objectives, and using guiding questions [18, 21]. The mouse/cursor is an example of a signal, and it can be visualized by the student when screen-capture vodcast software is used. Since the cursor cannot be seen by the user with Flash™-based vodcast software, faculty can animate arrows into their presentations to serve as signals to learners. Another potential solution to cognitive overload is to physically segment vodcasts or incorporate breaks (pauses) between successive segments of a presentation. Breaks help restore attention [35], and during a pause the learner can organize and integrate [21]. For screen-capture software, pauses can be incorporated for review questions prior to moving on to the next section of a vodcast; students can choose to pause or come back later to work on the questions. With Flash™-based vodcast software, the user is allowed to click forward after completing a question.

#### Promoting student engagement

If students do not watch vodcasts, they cannot learn from them, so a critical aspect of vodcast development is to include elements that promote student engagement [17]. Examples of vodcast engagement elements include short length, rapid and enthusiastic narration, and high quality sound and images. Vodcasts with poor quality sound and images can be annoying to the user [10, 12], and students may be less inclined to view them. An important engagement strategy in progress at SOMA is to provide high quality microphones to all faculty.

#### Flash or screen-capture vodcast software?

Is there an ideal vodcast software and is it different for students who receive relatively few vs. many vodcasts in their curricula, or for novice vs. advanced learners? Is the cost and relative difficulty of creating vodcasts with physically interactive software worth it? Are learning outcomes with physically interactive vs. non-physically interactive vodcasts different? These are challenging questions in need of further study. Although students in the residential learning environment had a preference for interactive practice questions, our learner preferences overall are in line with screen-capture software, which has lower demands on institutional cost and faculty time and effort. It is worth noting that presenters using screen-capture vodcast software can solicit interaction by, for example, posing questions/problems and asking students to pause and consider solutions, and by embedding links to interactive cases/games/problems.

### Limitations

This research is limited by the institutional and cultural contexts in which it was conducted. Surveys were collected anonymously in order to reduce the likelihood of response bias. The response rate varied for the three cohorts, and we were not able to characterize non-responders, who may or may not have had different perceptions of vodcasts. The three-week pilot course for the Class of 2019 (year 1 students) more than likely influenced perceptions. Length of time in medical school may also have influenced perceptions. The results of this study might not be generalizable to other educational programs or cultures that place less value on online learning. In attempting to apply these methods and findings in a different context, investigators should consider the specific constraints, type of online learning, outcome measures used, and the natural environment of the study setting.

## Conclusions

Analysis of survey results from three medical student cohorts yielded important information to guide vodcast creation and production improvements. Notable differences between students in residential and distance/blended learning environments were the preference for interactive practice questions for students in the residential setting, and a focus on efficiency for students in the blended learning environment. All interactive vodcast features were less valued by students in the blended learning environment. Possible explanations for these differences include the relative volume of vodcasts received, time available in the curriculum to view vodcasts, novice vs. advanced learners, and the potential for a vodcast novelty effect in year 1. The feedback gathered from this survey will be useful for both current and new faculty, and other academic institutions using vodcasts.

## Additional files

Additional file 1: Vodcast survey. (DOCX 320 kb)

Additional file 2: Summary of student responses to the prompt *Drag and drop each of the following vodcast attributes into the box that best describes their value to your learning: Essential, Nice to have, or Not helpful.* This figure shows the relative ranking of attributes that students deemed *Not helpful.* (DOCX 16 kb)

Additional file 3: Summary of student responses to the prompt *Drag and drop each of the following vodcast attributes into the box that best describes their value to your learning: Essential, Nice to have, or Not helpful.* This figure shows the relative ranking of attributes that students deemed *Nice to have* (DOCX 16 kb)

Additional file 4: Summary of student responses to the open-ended prompt *I prefer this length because*…*Other. Please explain.* (XLSX 14 kb)

Additional file 5: Summary of student responses to the open-ended prompt *What is your favored external (outside of SOMA) resource for vodcasts?* (XLSX 10 kb)

## Abbreviations

ATSU SOMA: A.T. Still University School of Osteopathic Medicine in Arizona
CHCs: Community health centers
OMS1s: Year 1 osteopathic medical students
OMS2s: Year 2 osteopathic medical students
OMS3s: Year 3 osteopathic medical students
