# Supplementary figures and images for: A descriptive, cross-sectional study of medical student preferences for vodcast design, format and pedagogical approach
Source: BMC Med Educ. 2017 May 19;17:89. doi: 10.1186/s12909-017-0926-z (PMC5438517; doi:10.1186/s12909-017-0926-z)

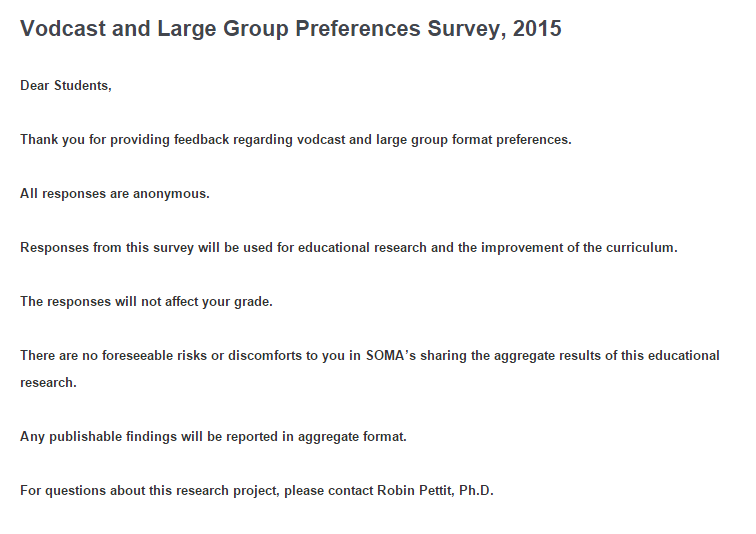


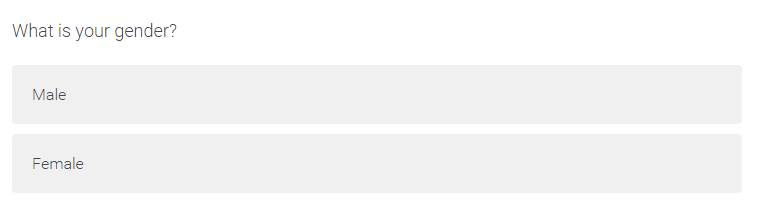


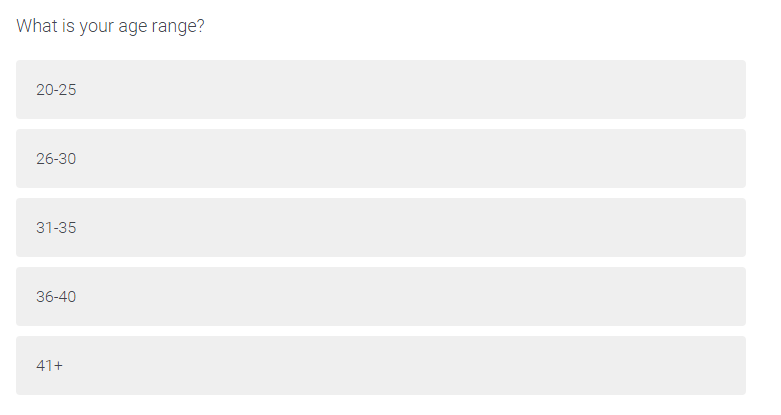


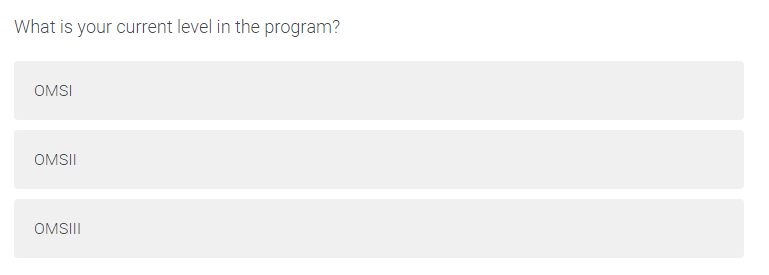


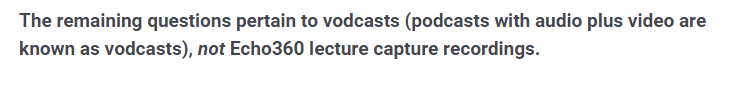


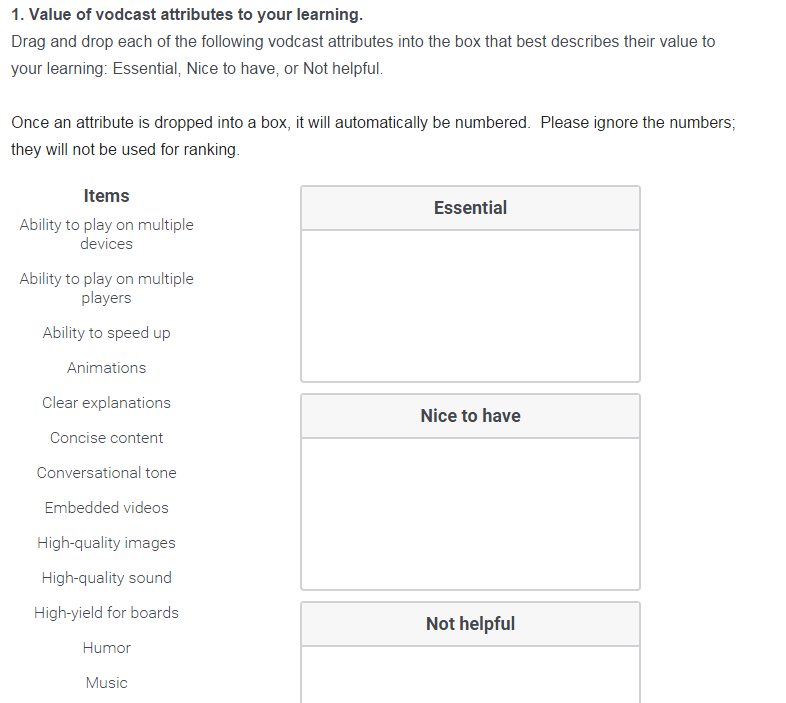


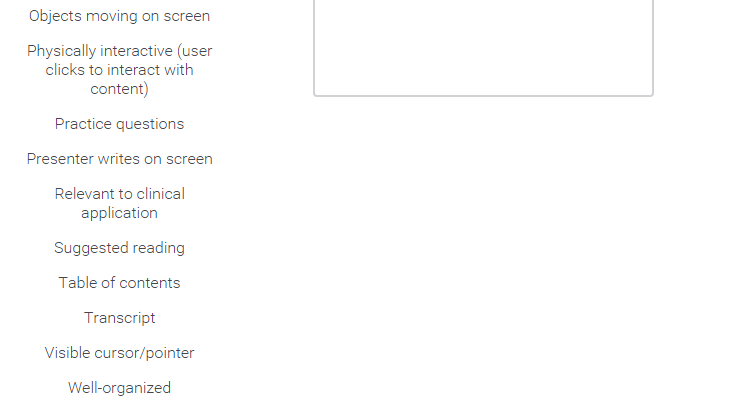


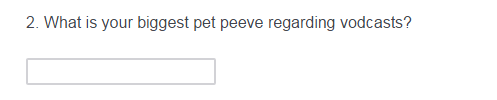


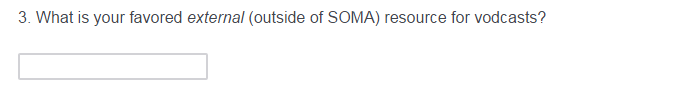


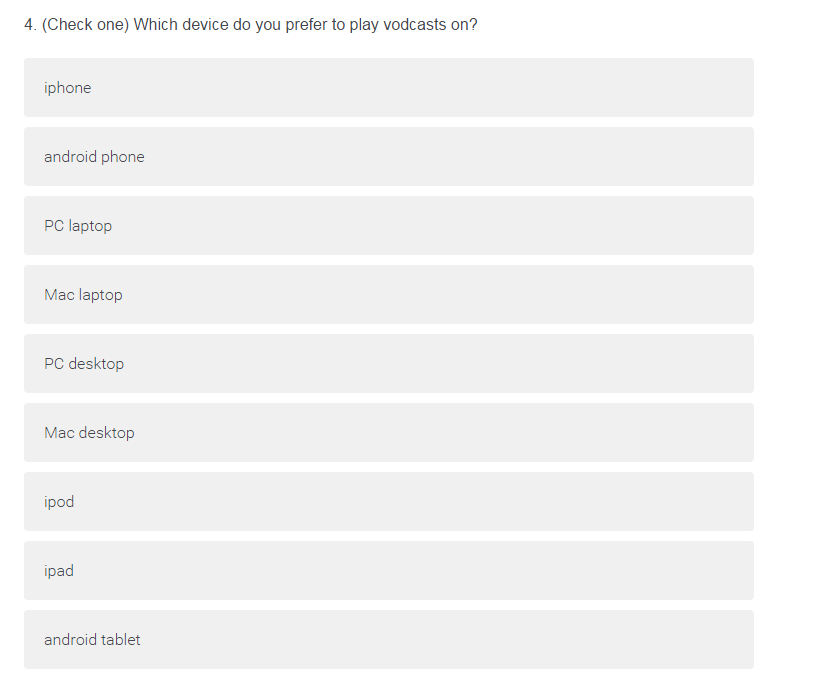


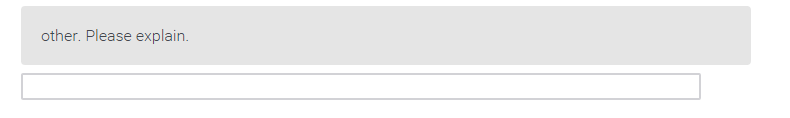


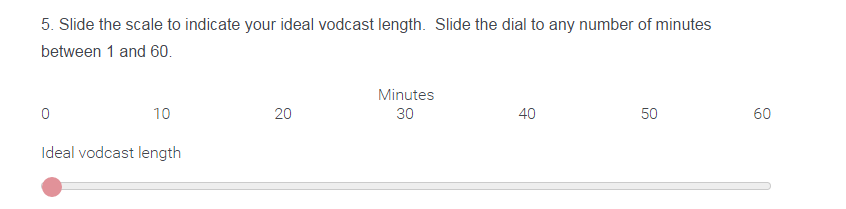


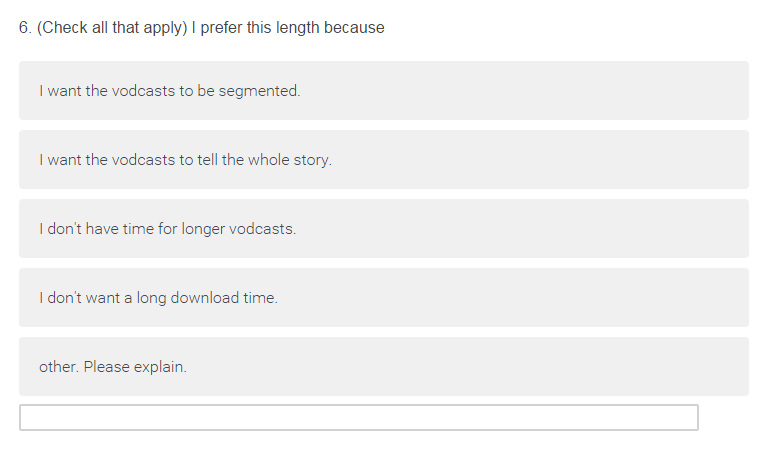


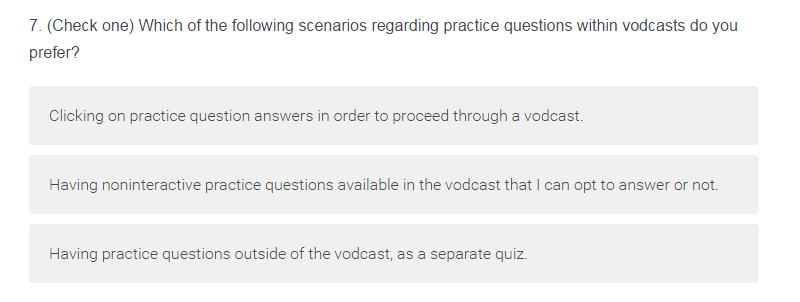


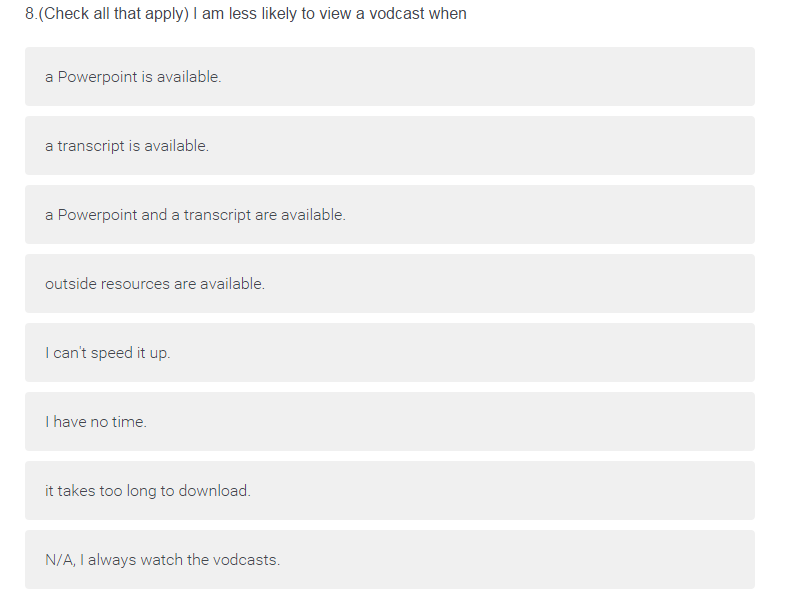

Supplement: Supplementary file 1 — Vodcast survey. (DOCX 320 kb) [file 12909_2017_926_MOESM1_ESM.docx]
