# Supplementary material for: A descriptive, cross-sectional study of medical student preferences for vodcast design, format and pedagogical approach
Source: BMC Med Educ. 2017 May 19;17:89. doi: 10.1186/s12909-017-0926-z (PMC5438517; doi:10.1186/s12909-017-0926-z)
Supplement: Supplementary file 2 [file 12909_2017_926_MOESM2_ESM.docx]

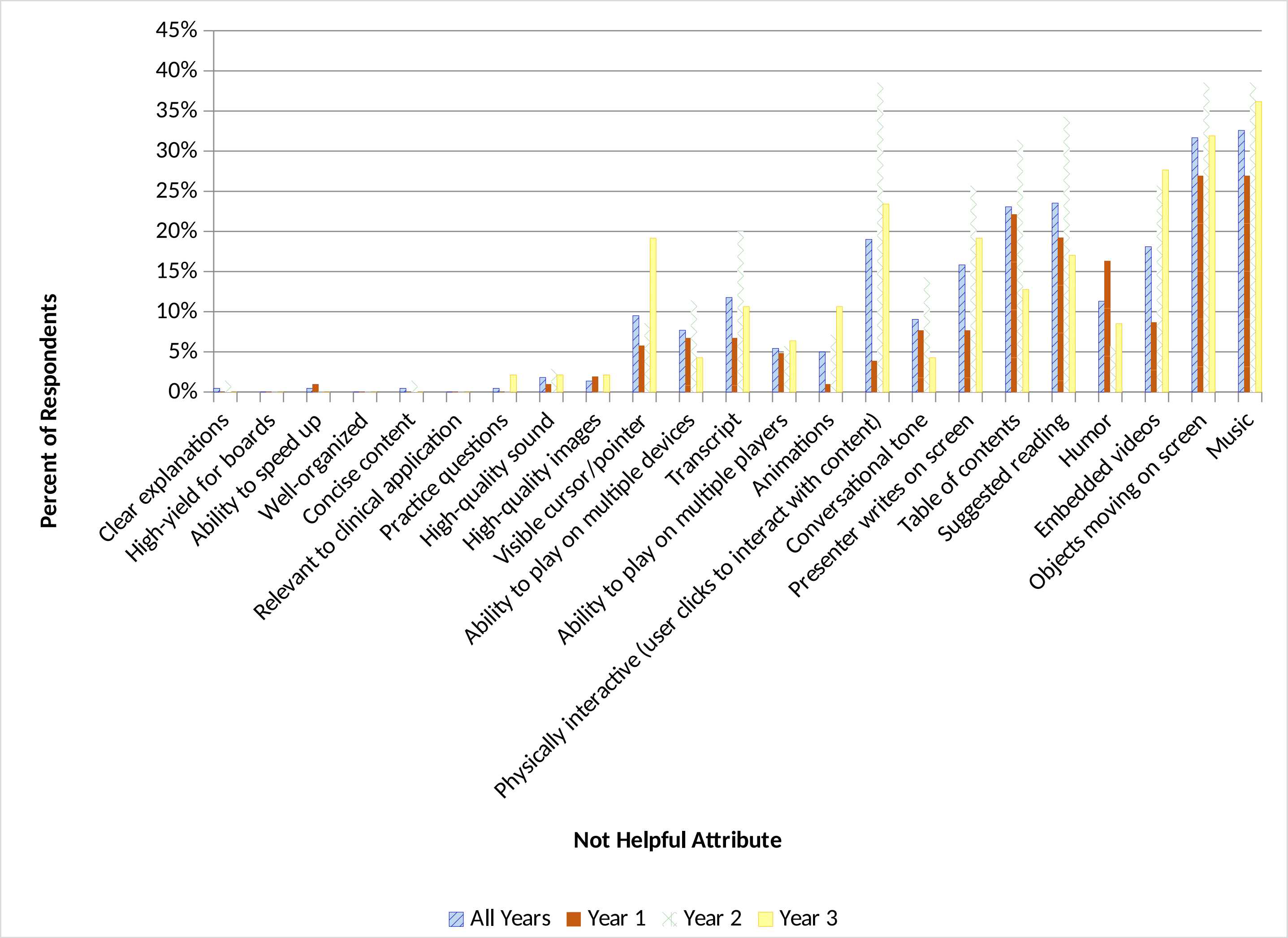
Percent of Respondents
45%
40%
35%
30%
25%
20%
15%
10%
5%
0%
Clear explanations
High-yield for boards
Ability to speed up
Well-organized
Concise content
Relevant to clinical application
Practice questions
High-quality sound
High-quality images
Visible cursor/pointer
Ability to play on multiple devices
Transcript
Ability to play on multiple players
Animations
Physically interactive (user clicks to interact with content)
Conversational tone
Presenter writes on screen
Table of contents
Suggested reading
Humor
Embedded videos
Objects moving on screen
Music
Not Helpful Attribute
All Years
Year 1
Year 2
Year 3
